# Brain Activity Associated With Regulating Food Cravings Predicts Changes in Self-Reported Food Craving and Consumption Over Time

**DOI:** 10.3389/fnhum.2020.577669

**Published:** 2020-11-12

**Authors:** Nicole R. Giuliani, Danielle Cosme, Junaid S. Merchant, Bryce Dirks, Elliot T. Berkman

**Affiliations:** ^1^Department of Special Education and Clinical Sciences, Prevention Science Institute, University of Oregon, Eugene, OR, United States; ^2^Communication Neuroscience Lab, Annenberg School for Communication, University of Pennsylvania, Philadelphia, PA, United States; ^3^Developmental Social Cognitive Neuroscience Lab, Neuroscience and Cognitive Science Program, Department of Psychology, University of Maryland, College Park, College Park, MD, United States; ^4^Brain Connectivity and Cognition Lab, Department of Psychology, University of Miami, Miami, FL, United States; ^5^Social and Affective Neuroscience Lab, Department of Psychology, Center for Translational Neuroscience, University of Oregon, Eugene, OR, United States

**Keywords:** food cue reactivity, food craving regulation, healthy food, unhealthy food, food craving, food consumption, brain activity

## Abstract

Neural patterns associated with viewing energy-dense foods can predict changes in eating-related outcomes. However, most research on this topic is limited to one follow-up time point, and single outcome measures. The present study seeks to add to that literature by employing a more refined assessment of food craving and consumption outcomes along with a more detailed neurobiological model of behavior change over several time points. Here, a community sample of 88 individuals (age: *M* = 39.17, *SD* = 3.47; baseline BMI: *M* = 31.5, *SD* = 3.9, range 24–42) with higher body mass index (BMI) performed a food craving reactivity and regulation task while undergoing functional magnetic resonance imaging. At that time—and 1, 3, and 6 months later—participants reported craving for and consumption of healthy and unhealthy foods via the Food Craving Inventory (FCI) and ASA24 (N at 6 months = 52–55 depending on the measure). *A priori* hypotheses that brain activity associated with both viewing and regulating personally desired unhealthy, energy-dense foods would be associated with self-reported craving for and consumption of unhealthy foods at baseline were not supported by the data. Instead, regression models controlling for age, sex, and BMI demonstrated that brain activity across several regions measured while individuals were regulating their desires for unhealthy food was associated with the self-reported craving for and consumption of healthy food. The hypothesis that vmPFC activity would predict patterns of healthier eating was also not supported. Instead, linear mixed models controlling for baseline age and sex, as well as changes in BMI, revealed that more regulation-related activity in the dlPFC, dACC, IFG, and vmPFC at baseline predicted decreases in the craving for and consumption of healthy foods over the course of 6 months.

## Introduction

For many of us, simply seeing or smelling something that reminds us of a favorite food can elicit a strong desire for that food. Strong desires, called cravings, often lead to the consumption of that food (e.g., Fedoroff et al., 2003). This frequently occurs for unhealthy or energy-dense foods like snacks and desserts (Massicotte et al., 2019). Consumption of such energy-dense foods is associated with increased risk for overweight [25 kg/m^2^ ≤ body mass index (BMI) < 30 kg/m^2^], obesity (BMI ≥ 30 kg/m^2^), and health consequences associated with higher body weight (Pereira et al., 2005; Duffey et al., 2007; Centers for Disease Control and Prevention, 2011).

Cravings are a form of “food cue reactivity,” or conditioned responses to food that are frequently accompanied by a suite of experiential, physiological, and neural responses (Boswell and Kober, 2016). However, people also work to regulate the cravings they experience, such that they don’t automatically lead to consuming the craved food (Giuliani and Berkman, 2015). A growing body of research has focused on brain activity associated with food cue reactivity and the regulation of these responses, with the goal of explaining how these neural responses predict subsequent health outcomes. At present, this research is mostly limited to studies with one follow-up time point, or a single outcome measure (e.g., BMI, snack consumption; Yokum et al., 2011; Lawrence et al., 2012). The present study seeks to extend this work by (1) employing a more refined assessment of food preference, craving, and consumption outcomes above and beyond BMI, (2) interrogating these patterns for both healthy and unhealthy foods, and (3) creating a more detailed model of behavior change over multiple time points.

### Behavior Change From Food Cue Reactivity

The subjective experience of craving predicts subsequent eating behavior in the presence (Cornell et al., 1989; Fedoroff et al., 1997, 2003; Nederkoorn et al., 2000) and absence of actual food cues (Gilhooly et al., 2007; Martin et al., 2008; Batra et al., 2013; Cleobury and Tapper, 2014; Crowley et al., 2014). Recent work suggests that this effect is specific to highly palatable energy-dense foods (Massicotte et al., 2019), which are also those that individuals most often report craving (Chao et al., 2014). Craving is related to but separable from liking; where craving implies a motivational state, liking is the hedonic reaction to the pleasure of the reward (Berridge, 2009). As such, a full investigation of the patterns of behavior change from food cue reactivity should interrogate both subjective liking for foods as well as craving for and intake of those foods.

Similarly, physiological and neural responses to food cues are also prospectively associated with both eating behavior and weight gain (Rogers and Hill, 1989; Nederkoorn et al., 2000; Nederkoorn and Jansen, 2002; Jansen et al., 2003; Stice et al., 2010; Yokum et al., 2011, 2014; Demos et al., 2012; Lawrence et al., 2012; Mehta et al., 2012; Murdaugh et al., 2012; Lopez et al., 2014; Winter et al., 2017; Versace et al., 2018). This literature, while robust, is not uniform; several studies investigating measures of food cue reactivity have failed to demonstrate associations with eating behavior and/or weight, or found an association with physiological cue reactivity but not self-reported reactivity (see Boswell and Kober, 2016). A recent meta-analysis by Boswell and Kober (2016) found a medium effect of food cue reactivity and craving on food consumption and weight (*r* = 0.33), which did not vary based on the presence or absence of an actual food cue to induce the craving.

Interestingly, the bulk of the neurobehavioral work in this area has focused on brain regions associated with reward processing and incentive valuation, including the ventral striatum (VS), ventromedial prefrontal cortex (vmPFC), and amygdala (Giuliani et al., 2018). Craving is thought to recruit more of this reward network than liking, but the overlapping neural structures make experimentally separating the two processes quite difficult (Havermans, 2011). Recent evidence suggests that liking may be more associated with BMI than craving (Polk et al., 2017), and that food cue reactivity in the VS may be more strongly associated with food consumption than the vmPFC, which tracks with craving (Lawrence et al., 2012). Past work from our lab found that, independent of an individual’s motivational state, activity in a broad network of regions involved in food cue reactivity, including the parahippocampal gyrus, cingulate, inferior occipital gyrus, and anterior insula, predicted consumption of a personally desired unhealthy food, but only in individuals who were not dieting (Giuliani et al., 2015). Other studies have found that activity in the inferior frontal gyrus (IFG, a lateral prefrontal region typically implicated in inhibitory control) while viewing food cues was positively associated with weight loss several months later (Neseliler et al., 2019). Therefore, the field’s dominant focus on the mesolimbic dopamine system as the neural seat of food cue reactivity, craving, and consumption may be obscuring the roles of other brain regions and complementary cognitive processes such as liking in predicting these behaviors.

### Behavior Change From Food Craving Regulation

Another complementary cognitive process is regulation. When individuals view energy-dense foods that they crave, they often don’t passively follow those temptations. People use a variety of methods to regulate cravings for things that aren’t in line with their long term goals (Giuliani et al., 2018). In the face of a tempting food, individuals engage in a variety of food craving regulation strategies. One popular strategy is reappraisal, which entails recontextualizing the food stimulus to change one’s response to it (e.g., focusing on the negative consequences of eating a desired but unhealthy food; Giuliani and Berkman, 2015). Indeed, the tendency and capacity to engage in food craving regulation varies substantially among individuals (Lowe et al., 2019), supporting the notion that obesity, overweight, and the consumption of unhealthy foods is much more complex than differences in cue reactivity and craving. Behavioral measures of food craving regulation have been found to predict changes in the consumption of both healthy and unhealthy food over time (Giuliani et al., 2015; Reader et al., 2018).

Regulating the motivation to consume a desired unhealthy food relies heavily on the prefrontal cortex (PFC), which is implicated in the control over human behaviors, actions, and thoughts. When individuals are instructed to reappraise or suppress their cravings for food, increased activity in the dorsolateral PFC (dlPFC), dorsal anterior cingulate cortex (dACC), and inferior temporal lobe is regularly observed (Giuliani et al., 2014; Han et al., 2018). A recent meta-analysis found that BMI is negatively associated with activity in the left dlPFC, right ventrolateral PFC (vlPFC), and IFG during regulation, which suggests that individuals with higher BMIs may be less able to recruit the lateral PFC to regulate their food choices (Han et al., 2018). Indeed, it is believed that lateral regions like the dlPFC modulate incentive valuation and reward processing within medial regions like the vmPFC and ventral striatum, which then affects the likelihood that individuals will select food items on the basis of healthiness compared to taste (Hare et al., 2009; Maier et al., 2015, though see Tusche and Hutcherson, 2018). Of these regions, the vmPFC is uniquely thought to support value-based decision-making (Berkman et al., 2017), in which choice options are assigned subjective values and a decision is made through a dynamic integration process of gains and costs. Assessments of vmPFC activation during decision-making, including in this study, typically take place before consumption as people consider their degree of motivation to approach (e.g., eat or purchase) the food stimuli. As such, vmPFC activation as measured by tasks such as the one used in this study can be considered an index of “wanting” as opposed to “liking.”

Moving laterally, further evidence for the role of the lateral PFC in regulation comes from experimental manipulation of dlPFC activity. Stimulation of this region attenuates food cravings and the consumption of energy-dense foods in individuals who report strong food cravings, and inhibiting dlPFC activity is associated with overconsumption of energy-dense foods via changes in inhibitory control (Lowe et al., 2019). While there is considerable evidence linking eating behavior to brain activity (see Giuliani et al., 2018), additional evidence supports the effect of weight on brain function. Recent work on individuals who underwent bariatric surgery revealed that weight loss resulted in significant increases in dlPFC activity during food appraisal (Holsen et al., 2018). As such, this literature suggests a strong but bidirectional association between lateral PFC activity and eating behavior. Thus, according to Lowe et al. (2019), weaker lateral PFC responses to food cues may increase individual susceptibility to the rewarding properties of energy-dense foods, which prompts overconsumption, which then, over time, leads to lateral PFC dysregulation, exacerbated sensitivity to food rewards, and increased adiposity. While this model is intriguing, few studies have attempted to reconcile this PFC-centered model with the large literature implicating mesolimbic food cue reactivity in predicting food craving and consumption or the recent work indicating that both reactivity and regulation information are integrated in the vmPFC.

### The Present Study

The present study aimed to address this gap by measuring brain activity associated with both food cue reactivity and regulation, and prospectively investigating how it predicted changes in self-reported liking of, craving for, and consumption of both healthy and unhealthy foods over the course of 6 months in a sample of middle-aged adults with higher BMIs.

First, we hypothesized that brain activity associated with both viewing and regulating personally desired unhealthy, energy-dense foods would be associated with self-reported craving and consumption of unhealthy foods at baseline. We also explored the association between brain activity and liking of, craving for, and consumption of healthy foods in order to examine the specificity of this effect to unhealthy food. Second, we hypothesized that activity in the vmPFC during the viewing of unhealthy foods would mediate the association between regulation-related brain activity and self-reported craving for and/or consumption of these foods. Third, we hypothesized that value-related brain activity in the vmPFC during food craving regulation at baseline would predict a decrease in the consumption of unhealthy food and an increase in the consumption of healthy food over 6 months. We also explored the role of other brain regions in predicting eating behavior change.

## Materials and Methods

### Participants

A community sample of 105 middle-aged individuals with higher BMIs who intended to eat more healthfully were screened and enrolled into a larger project investigating a text message-based intervention aimed to improve eating habits. Inclusion criteria for the overarching study included (1) an approximate BMI between 25–40; (2) early, middle-age (i.e., approximately 35–45 years old); (3) no psychiatric, neurological, or eating disorders; (4) no fMRI contraindications; (5) not actively enrolled in a diet program or any other type of eating intervention; and (6) self-reported desire to eat more healthfully. Ten participants were excluded for non-compliance or repeatedly missing appointments, or decided to drop out before completion of the baseline session. A further 7 participants were excluded from the MRI analyses because they had excessive motion artifacts (defined below; *n* = 2), were not compliant with enrollment criteria (*n* = 2), had structural anomalies (*n* = 1), or for whom timing data were lost due to a technical error (*n* = 2). Therefore, analyses for this study are from the 88 individuals who provided useable MRI data (age: *M* = 39.2, *SD* = 3.57, range 33–46; baseline BMI: *M* = 31.5, *SD* = 3.9, range 24–42); they did not differ with regard to age or BMI from those who provided MRI data that were not useable. These participants were 81.91% female, and 79.5% identified as Caucasian, 8% Hispanic, 3.4% Black (not of Hispanic origin), 2.3% Middle Eastern, 1.1% each South Asian and American Indian/Alaskan Native, and 3.4% other.

An *a priori* power analysis was not conducted for this study; sample size was determined by budget and the duration of the grant award period. A *post hoc* power analysis in G^∗^Power (Faul et al., 2009) indicated that we were adequately powered to test for medium-sized brain-behavior associations at baseline (f^2^ = 0.156, with power = 0.8, α = 0.05, 5 predictors). With regard to change over time, we achieved up to 89% power (time x dlPFC interaction predicting craving for healthy foods) according to *post hoc* power simulations using simr (Green and MacLeod, 2016). However, many of the other findings were underpowered and should be interpreted accordingly. Hypotheses and analytical procedures for this study were preregistered at the Open Science Framework^1^.

### Protocol

Participants were recruited for this study using a combination of online, newspaper, and public advertising. Interested individuals were screened for exclusion criteria via phone, and eligible participants were sent a battery of measures to complete before arriving for their baseline session. Participants were scheduled for their baseline lab visit, and asked to complete the pre-baseline measures within 24 h of the visit. Pre-baseline measures included demographic information, assessments of eating behavior, self-reported food craving and liking, and a set of psychometric measures not presented here. Participants were instructed to not eat anything for at least an hour before the baseline visit. During the baseline (T1) visit, participants provided informed consent, after which they were screened for MRI contraindications and instructed on the requirements of the study. Weight and height were measured, after which participants took part in an MRI session (described below). After the MRI session, they were randomized into intervention and control groups. The intervention, in which participants received self-authored text messages aligning healthy eating with personal values for 28 days after T1, did not have a significant effect on changes in BMI, healthy eating, or food craving compared to the control condition, where participants received standardized healthy eating messages. For the purposes of the analyses pursued here of measures acquired after randomization to condition, individuals were collapsed across groups, with group assignment controlled in the statistical models as a dummy-coded variable.

After the 28 days follow-up period, participants returned to the lab where they repeated all baseline measures with the exception of demographics and the MRI session (T2). Eating behavior was assessed over two separate days in advance of the visit. Three months after baseline (T3), participants were emailed instructions as to how to provide information on eating behavior and self-reported food craving and liking online. Six months after baseline (T4), participants returned to the lab where they repeated the procedures of the T2 visit.

### Measures

#### Body Mass Index (BMI)

Weight and height were measured using a commercially available weight scale and wall ruler, and BMI was calculated by dividing the participant’s weight in kilograms by height in square meters. These measures were collected in the laboratory at T1, T2, and T4. The number of participants from whom we collected this information at each time point is listed in Table 1.

**TABLE 1:** Demographic and self-report information.

Measure	T1—0 months		T2—1 month		T3—3 months (online)		T4—6 months	
	*M* (*SD*)/% (*n*)	N	*M* (*SD*)	N	*M* (*SD*)	N	*M* (*SD*)	N
Age	39.2 (3.57)	88						
Gender (# female)	83.9%(73)							
BMI	31.5 (3.9)	88	31.57 (3.92)	78			31.18 (4.48)	52
FCI—healthy like rating	2.67 (0.46)	86	2.61 (0.45)	74	2.55 (0.47)	57	2.53 (0.50)	53
FCI—healthy crave rating	2.14 (0.73)	88	1.97 (0.68)	77	1.99 (0.68)	59	1.90 (0.63)	56
FCI—unhealthy like rating	2.70 (0.39)	86	2.61 (0.41)	74	2.63 (0.41)	57	2.61 (0.45)	53
FCI—unhealthy crave rating	2.47 (0.57)	88	2.20 (0.58)	77	2.26 (0.55)	59	2.16 (0.57)	56
ASA24—total kcal consumed	2087.94 (790.44)	87	1827.13 (688.58)	78	1805.44 (680.91)	55	1790.07 (797.25)	55
ASA24—HEI	52.86 (12.75)	87	50.75 (11.99)	77	50.38 (13.20)	55	54.24 (11.92)	55
ASA24—fruit/vegetable consumption	2.61 (1.28)	87	2.63 (1.28)	77	2.47 (1.26)	55	2.88 (1.09)	55
ASA24—empty calorie consumption	11.67 (5.22)	87	11.48 (5.73)	78	11.68 (5.19)	55	12.91 (5.45)	55

*Age and gender were only collected at baseline; BMI was collected in person at baseline, T2, and T4; all other variables were collected at every time point. BMI = Body Mass Index; FCI = Food Craving Inventory; HEI = Healthy Eating Index.*

#### Food Craving Inventory (FCI)

The FCI (White et al., 2002) is a 67-item self-report measure of cravings for and liking of specific foods. Craving was defined as “an intense desire for a specific food that is difficult to resist.” Participants were asked to rate the frequency of cravings for the past 30 days on a 5-point Likert scale (1 = “not at all,” 5 = “nearly every day”). They were also asked to rate how much they liked each food on a 4-point Likert scale (1 = “dislike,” 4 = “love”).

Foods were grouped into categories and ratings in each category were averaged for craving and liking separately. The foods included in each category are listed in the Supplementary Materials. We created composite measures of liking of and craving for unhealthy foods by averaging across ratings in the following categories: processed meats, fried foods, red meats, sugary foods, and empty calories. The same procedure was followed for healthy foods with the following categories: vegetables, fruits, lean proteins, whole grains, and olive oil.

#### ASA24

Eating behavior was assessed via the Automated Self-Administered 24 h (ASA24^®^) Dietary Assessment Tool (Subar et al., 2012) which allows for the calculation of the Healthy Eating Index (HEI; Guenther et al., 2013) using calculations provided by the developers of the ASA^2^. Following the recommendations proposed by the developers of this measure, participants completed the ASA twice at each time point to obtain a more representative estimate of daily eating behavior. We used the HEI as our measure of healthy eating as it quantifies how well a person’s eating behavior aligns with recommended dietary guidelines for Americans. We also created an average of HEI indices 1–4, which captured daily consumption of total vegetables, greens and beans, total fruit, and whole fruit. In addition, we used the ASA24’s calculation of total kilocalories consumed and empty calories consumed. All data were averaged across the 2 days of data collection at each time point.

#### Regulation of Craving (ROC) Task

Participants were trained to decrease their desire to consume personally-desired (for task purposes these are referred to as “craved” foods) foods using cognitive reappraisal (Giuliani et al., 2014; Giuliani and Pfeifer, 2015). Participants viewed unhealthy craved foods (“Craved” condition), unhealthy not-craved foods (“Not Craved” condition), or healthy vegetables (“Neutral” condition). For unhealthy craved foods, participants either actively viewed the foods (“Look” condition) or reappraised their craving for them (“Regulate” condition). On “Look” trials, participants imagined how they would interact with the food if it were in front of them. On “Regulate” trials, participants reappraised the foods by focusing on the short- or long-term negative health consequences associated with consumption (e.g., stomach aches, weight gain, cavities, etc.). Participants generated several negative health consequences with the help of the experimenter, to ensure they had multiple reappraisals they could use during the task. To minimize demand characteristics (e.g., reduced craving ratings on regulate trials), participants were reassured they were not expected to be able to regulate well on every trial and were told that it was important to rate their cravings honestly. Neutral stimuli were only viewed under “Look” instructions, and are not used in the present analyses. To keep task time to a minimum, only craved foods were viewed under “Regulate” instructions.

**FIGURE 1:**
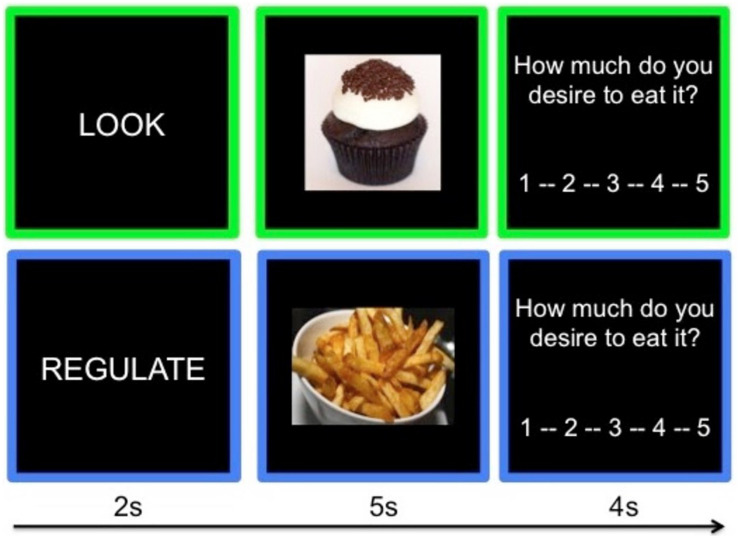
Example trials from the ROC task.

To maximize craving, participants selected their most craved and least craved food from the following menu of unhealthy food categories for the “Crave” and “Not Craved” conditions respectively: chocolate, cookies, donuts, French fries, ice cream, pasta, pizza. Stimuli were independently rated for desirability, such that the mean desirability of stimuli within each unhealthy food category did not differ significantly across categories (Giuliani et al., 2013). As such, each participant viewed a set of individually adapted unhealthy food stimulus categories; across all participants, the only domain on which the “Craved” and “Not Craved” conditions differed was with regard to individual food preferences. Each condition (Look Neutral, Look Craved, Look Not Craved, and Regulate Craved) had 20 trials, presented across two task runs. On each trial (see Figure 1), participants are presented with an instruction (2 s; Look or Regulate), viewed a food image while following the instruction (5 s), and rated their craving for the food on a 5-point Likert scale (4 s; 1 = not at all, 5 = very much). Each 11s trial of this event-related design was followed by a jittered fixation cross (*M* = 1 s) and trial order is optimized using a genetic algorithm (Wager and Nichols, 2003). Stimuli were presented using Psychtoolbox 3 (Brainard, 1997), and participants responded using a five-button box.

### Neuroimaging Data Acquisition and Preprocessing

Neuroimaging data were acquired on a 3T Siemens Skyra scanner at the University of Oregon Lewis Center for Neuroimaging. We acquired a high-resolution anatomical T1-weighted MP-RAGE scan (TR/TE = 2,500.00/3.43 ms, 256 × 256 matrix, 1 mm thick, 176 sagittal slices, FOV = 208 × 208 mm), functional images with a T2^∗^- weighted echo-planar sequence (72 axial slices, TR/TE = 2,000.00/27.00 ms, 90-degree flip angle, 100 × 100 matrix, 2 mm thick, FOV = 208 × 208 mm, multiband acceleration factor = 3), and opposite phase encoded echo-planar images to correct for magnetic field inhomogeneities (72 axial slices, TR/TE = 6,390.00/47.80 ms, 90-degree flip angle, 104 × 104 matrix, 2 mm thick, FOV = 208 × 208 mm).

Neuroimaging data were preprocessed using fMRIPrep 1.1.4 (Esteban et al., 2019). A detailed account of the preprocessing pipeline appears in Supplementary Material, but briefly, anatomical images were segmented and normalized to MNI space using FreeSurfer (Fischl, 2012); functional images were susceptibility distortion corrected, realigned, and coregistered to the normalized anatomical images. Normalized functional data were then smoothed (6 mm FWHM) in SPM12 (Wellcome Department of Cognitive Neurology^3^).

### Neuroimaging Analysis

Event-related condition effects were estimated in first-level analyses using a fixed-effects general linear model and convolving a canonical hemodynamic response function to stimulus events using SPM12. Regressors were entered for each experimental condition (regulate craved, look craved, look not-craved, look neutral) and were modeled during stimulus presentation (5 s). Additional regressors of no interest were added for the instruction and rating periods. Five motion regressors were modeled as covariates of no interest. Realignment parameters were transformed into Euclidean distance for translation and rotation separately; we also included the displacement derivative of each, resulting in a total of four motion regressors. Another regressor of no interest indicated images with motion artifacts (e.g., striping) identified via automated motion assessment (Cosme et al., 2018) or visual inspection. Data were high-pass filtered at 128s and temporal autocorrelation was modeled using FAST (Corbin et al., 2018). Task runs with > 10% of volumes contaminated with motion artifacts were excluded (*n* = 8) or if they were missing 80% or more responses (*n* = 5). Participants were excluded completely if both task runs were excluded (*N* = 4). For each participant, we computed the following linear contrasts: Look Craved > Look Not Craved (LC > LNC, called “reactivity”) and Regulate Crave > Look Crave (RC > LC, called “regulation”) using SPM12.

We estimated second-level random effects for each contrast using a one-sample *t*-test in SPM12. Multiple comparisons were corrected using cluster-extent thresholding implemented in AFNI version 18.2.04 (Cox, 1996). In accordance with recent guidelines (Cox et al., 2017), the spatial autocorrelation function was first estimated for each subject and task run separately using AFNI’s 3dFWHMx, and then averaged across subjects. To determine probability estimates of false-positive clusters given a random field of noise, Monte-Carlo simulations were conducted with AFNI’s 3dClustSim using the average autocorrelation across subjects (ACF = 0.70, 4.27, 8.63). For each model, a voxel-wise threshold of *p* < 0.001 and cluster extent of k = 61 was estimated (voxel dimensions = 2 × 2 × 2 mm) to achieve a whole-brain familywise error rate of α = 0.05.

### ROI Definition and Extraction

Region of interests were defined anatomically due to the *a priori* nature of our hypotheses. The VS and vmPFC ROIs were from Bartra and colleagues’ (2013) publicly available database of ROIs based on a 200 paper meta-analysis of subjective value (Bartra et al., 2013)^4^. The dlPFC, IFG, and dACC ROIs were all created using the WFU PickAtlas (Tzourio-Mazoyer et al., 2002; Maldjian et al., 2003). For the dlPFC ROI, we selected Brodmann areas 8 and 9, and dilated the mask by 1mm. The IFG ROI was defined using the WFU PickAtlas TD Labels inferior frontal gyrus ROI. The dACC ROI was created by bounding the WFU PickAtlas TD Labels anterior cingulate ROI on the y-axis at 0–32. Per our hypotheses, we extracted individual parameter estimates from the VS and vmPFC ROIs from the LC > LNC contrast, and dlPFC, IFG, and dACC ROIs from the RC > LC contrast. These three regulation ROIs were averaged and combined into a composite for analysis purposes, and also explored separately.

In addition, we explored associations between regulation-related brain activity and food craving (from the FCI) and consumption (from the ASA24) by creating 6 mm^3^ spherical ROIs around the peaks from the RC > LC contrast (listed in Table 2, labeled with an ^∗^). The purpose of these ROIs was to explore the association of regulation-related brain activity with longitudinal patterns of healthy and unhealthy food liking, craving, and consumption orthogonal to the contrast that defined the ROIs. These seven ROIs were averaged and combined into a composite to reduce the number of statistical tests run, and also explored separately.

**TABLE 2:** Correlations at baseline (T1).

Variable	1	2	3	4	5	6	7	8
1. BMI								
2. FCI—healthy like rating	–0.040							
3. FCI—healthy crave rating	0.207	0.467**						
4. FCI—unhealthy like rating	0.052	0.335**	0.154					
5. FCI—unhealthy crave rating	0.260**	0.005	0.488**	0.592**				
6. ASA24—total kcal consumed	–0.070	–0.010	–0.169	0.263*	0.077			
7. ASA24—HEI	0.031	0.128	0.075	–0.141	–0.093	–0.071		
8. ASA24—fruit/vegetable consumption	0.035	0.148	0.088	–0.071	–0.078	–0.056	0.680**	
9. ASA24—empty calorie consumption	0.168	–0.038	–0.003	−0.221*	–0.088	−0.322*	0.578**	0.160

BMI, body mass index; FCI, Food Craving Inventory; HEI, healthy eating index. *Indicates p < 0.05. ** indicates p < 0.01.

### Statistical Analyses

Statistical analyses were conducted in R (version 3.6.1; R Core Team, 2019). First, variables were investigated for skewness and kurtosis; all exhibited acceptable distributions and were retained for further analysis. We then created a series of multilevel models predicting liking, craving, and intake of healthy and unhealthy foods with time at Level 1 and person at Level 2 using lmer in R (Bates et al., 2015). Because the models failed to converge when including the linear effect of time (month slope) as a random effect, only participant intercepts were modeled as random effects. The time variable (number of months since T1) was centered at 0, and the rest of the variables were grand mean centered. These models included main effects of brain activity, BMI, age, gender, and condition at baseline, as well as the main effect of time and the interaction between time and brain activity. Lastly, we investigated the pattern of missingness in this data set using the non-parametric MCAR (Missing Completely at Random) test proposed by Jamshidian and Jalal (2010, as implemented in the MissMech R package, Jamshidian et al., 2014). Here, a significant result for the Hawkins test of normality and homoscedasticity indicates that the hypothesis of MCAR would be rejected, and results should be interpreted accordingly.

To interrogate hypothesis 1, that food craving reactivity and regulation brain activity would be associated with self-reported craving for and consumption of unhealthy food at baseline, we assessed these models for main effects of brain activity on FCI-reported craving for unhealthy foods and ASA24-reported consumption of empty calories. Because eight separate models (4 ROIs × 2 dependent variables) were run to address this hypothesis, a Bonferroni correction was used to correct for multiple comparisons. The corrected alpha was therefore set at *p* < 0.0063. Additional models assessing self-reported liking of unhealthy foods were also included, in order to separate out the motivational state (craving) from the hedonic response (liking). We also explored the associations between brain activity and the liking of, craving for, and consumption of healthy foods at baseline, in order to examine the specificity of previously reported brain-behavior associations by food type. Because these analyses were exploratory, we only report estimates and 95% confidence intervals for effects that surpass α < 0.05 in the text; all results are included in supplementary material available online^5^. To interrogate hypothesis 2, that vmPFC activity would mediate the relation between regulation-related brain activity and unhealthy food craving and/or consumption, we planned to run mediation models in R using lavaan (Rousseel, 2012) for any and all significant associations between regulation-related brain activity and behavior at baseline. These models would be constructed as follows: regulation-related brain activity would be the independent variable; reactivity-related vmPFC activity would be the mediator; the craving or consumption variable associated with regulation-related activity would be the dependent variable; and BMI, age, gender, and condition included as covariates. Lastly, to interrogate hypothesis 3, that vmPFC brain activity associated with regulating the desire for personally desired energy-dense foods at baseline would predict an increase in the consumption of healthy food and a decrease in unhealthy food over 6 months, we examined the time x brain interaction terms from the multilevel models. We additionally explored other reactivity- and regulation-related brain activity in other regions for this interaction on liking of, craving for, and consumption of both food types.

## Results

### Baseline

Baseline data from the FCI and the ASA24 are presented in Table 1, along with relevant demographic information. Correlations among non-brain variables at baseline are presented in Table 2. BMI was significantly positively correlated with self-reported craving for unhealthy [*r*(88) = 0.260, *p* = 0.014] foods. Liking and craving were significantly correlated for both healthy [*r*(88) = 0.467, *p* < 0.001] and unhealthy [*r*(88) = 0.592, *p* < 0.001] foods. Total calories consumed was positively correlated with self-reported liking of unhealthy foods [*r*(87) = 0.263, *p* = 0.015], and negatively correlated with consumption of empty calories [*r*(87) = −0.322, *p* = 0.002]. The ASA24’s HEI was positively correlated with fruit and vegetable consumption [*r*(87) = 0.680, *p* < 0.001] and empty calories [*r*(87) = 0.578, *p* < 0.001]; it was not significantly associated with liking or craving of healthy foods (*p*-values > 0.24). Consumption of empty calories was significantly negatively associated with self-reported liking of unhealthy foods [*r*(85) = −0.221, *p* = 0.042] and total calories consumed [*r*(87) = −0.322, *p* = 0.002].

**FIGURE 2:**
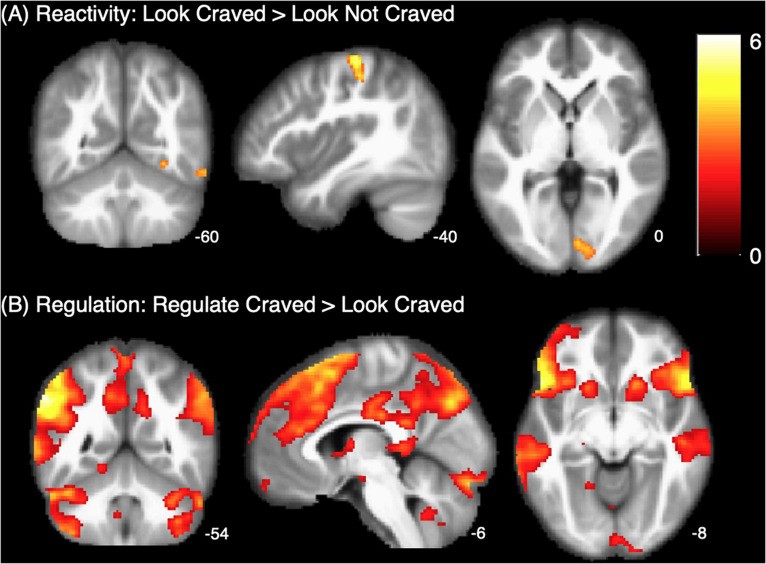
Whole brain maps of **(A)** reactivity and **(B)** regulation. *p* > 0.001, *k* = 61.

As shown in Figure 2A, whole brain results for the main effect of food cue reactivity revealed significant clusters in the left postcentral gyrus, right lingual gyrus, right fusiform gyrus, and right inferior temporal gyrus. Peaks and cluster sizes are listed in Table 3. The main effect of regulation was associated with a very large cluster of activity with subpeaks in the left supramarginal gyrus, right post-medial frontal gyrus, and left inferior gyrus. Additional peaks were located in the right supramarginal gyrus, midbrain, left cerebellum, and left parahippocampal gyrus (Figure 2B and Table 3).

**TABLE 3:** Regions, MNI coordinates, cluster sizes, and peak t values for the Look Craved > Look Not Craved and Regulate Craved > Look Craved main effects (*p* < 0.001, *k* = 61 threshold used for all contrasts).

Contrast and region	MNI Coordinates (x, y, z)	Cluster size	Peak t
**Reactivity: Look Craved > Look Not Craved**			
Left postcentral gyrus	(−46, −26, 64)	285	6.42
Right lingual gyrus	(6, −86, −2)	165	4.37
Right fusiform gyrus	(30, −60, −10)	84	4.14
Right inferior temporal gyrus	(58, −58, −16)	75	4.08
**Regulation: Regulate Craved > Look Craved**			
Left supramarginal gyrus*	(−60, −52, 40)	46,121	12.03
Right post-medial frontal gyrus*	(6, 12, 70)		10.83
Left inferior frontal gyrus*	(−52, 20, −6)		10.8
Right supramarginal gyrus*	(64, −46, 36)	3,180	8.34
Right middle temporal gyrus	(48, −30, −2)		7.89
Midbrain	(0, −18, −26)	79	5.61
Left cerebellum (IX)*	(−6, −56, −44)	149	4.49
Left parahippocampal gyrus*	(−10, −12, −18)	71	3.97

* mm^3^ sphere ROI created at that location.

In contrast to Hypothesis 1, reactivity-related activity in neither the VS ROI nor the vmPFC ROI was significantly associated with self-reported craving for or consumption of unhealthy foods at baseline. Average regulation-related brain activity in neither the *a priori* ROIs nor the peak ROIs were significantly associated with self-reported craving for or consumption of unhealthy foods. Full models are available in the supplementary material available online (see footnote).

Regulation-related activity in the average of the *a priori* ROIs was positively associated with consumption of fruits and vegetables, *B* = 0.73, 95% CI [0.08, 1.37], SE = 0.33, *t*(108.06) = 2.24, *p* = 0.027. Exploration of the individual ROIs revealed that this effect was only statistically significant in the dlPFC, *B* = 0.68, 95% CI [0.07, 1.28], SE = 0.31, *t*(108.96) = 2.21, *p* = 0.029. In addition, regulation-related activity in the vmPFC *a priori* ROI was positively associated with craving for healthy foods, *B* = 0.27, 95% CI [0.02, 0.52], SE = 0.13, *t*(90.80) = 2.11, *p* = 0.037. While the average of the peak ROIs from the RC > LC contrast was not significantly associated with liking, craving, or consumption, individual peaks showed intriguing associations with behavior at baseline. Left cerebellum activity (−6, −56, −44) was positively associated with the HEI, *B* = 4.18, 95% CI [0.64, 7.72], SE = 1.79, *t*(124.12) = 2.34, *p* = 0.021, and fruit and vegetable intake, *B* = 0.58, 95% CI [0.21, 0.95], SE = 0.18, *t*(109.81) = 3.13, *p* = 0.002. This pattern was also observed in the right supramarginal gyrus [64, −46, 36; HEI: *B* = 4.07, 95% CI [0.63, 7.52], SE = 1.74, *t*(123.28) = 2.34, *p* = 0.021; fruit and vegetable intake: *B* = 0.49, 95% CI [0.13, 0.85], SE = 0.18, *t*(110.40) = 2.72, *p* = 0.008]. Lastly, activity in the right post-medial frontal peak (6, 12, 70) showed an inverse relationship with the consumption of empty calories, *B* = −1.80, 95% CI [−3.50, −0.09], SE = 0.86, *t*(136.97) = −2.08, *p* = 0.039. Because Hypothesis 1 was not supported by the data, the criteria for assessing the mediation outlined in Hypothesis 2 was also not met.

### Change Over Time

As shown in Table 1, there was notable attrition over time. A test for the nature of the missing data revealed that these data were not Missing Completely At Random (Hawkins Test *p* < 0.001). However, follow-up group comparisons between participants who provided data at all three follow-up time points compared to those who did not indicated that these groups did not significantly differ with regard to age, gender, intervention condition, reactivity or regulation-related brain activity, or baseline BMI, healthy eating (both HEI and fruit/vegetable consumption), or liking/craving for healthy and unhealthy foods. The individuals who provided complete data did, however, report consuming significantly fewer calories per day at baseline compared to those who provided incomplete data, *F*(1, 85) = 4.61, *p* = 0.035. As such, the following results should be interpreted with this limitation in mind.

**FIGURE 3:**
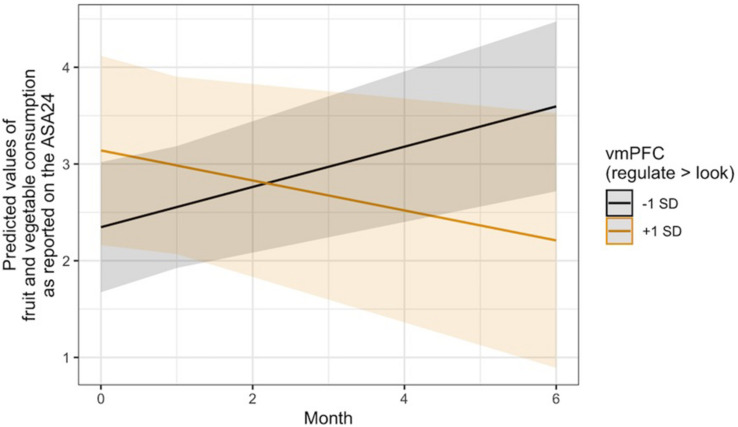
Visualization of the time x vmPFC activity interaction predicting changes in self-reported fruit and vegetable intake over time via the ASA24. Lines indicate the association at –1 (black) and + 1 (gold) SD from the mean vmPFC activity during Regulate Craved > Look Craved.

With regard to Hypothesis 3, linear mixed models controlling for changes in BMI and age, gender, and intervention condition indicated that regulation-related activity in the vmPFC ROI was not associated with changes in the consumption of unhealthy food over time, operationalized as self-reported intake of empty calories via the ASA24. It was, however, significantly associated with changes in fruit and vegetable intake over time, *B* = −0.11, 95% CI [−0.21, −0.00], SE = 0.05, *t*(137.64) = −2.03, *p* = 0.045. A visual interrogation of this effect revealed a crossover interaction (Figure 3), such that individuals who recruited more vmPFC activity during the regulation of the desire to consume unhealthy food reported consuming fewer fruits and vegetables over time, whereas those who displayed less vmPFC activity during regulation reported eating more fruits and vegetables.

**FIGURE 4:**
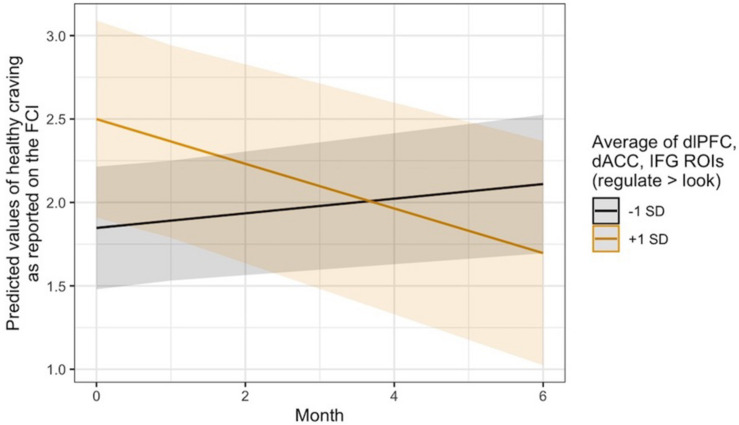
Visualization of the time x activity interaction predicting changes in self-reported craving for healthy foods over time via the ASA24. Lines indicate the association at –1 (black) and + 1 (gold) SD from the mean activity in the dlPFC, dACC, and IFG ROIs during Regulate Craved > Look Craved.

Exploratory analyses revealed that this same pattern occurred in thevmPFC with regard to changes in self-reported craving for healthyfoods, *B* = −0.04, 95% CI [−0.08, −0.01], SE = 0.02, *t*(124.48) = −2.36, *p* = 0.020. It was also seen in the average regulation-related brain activity across the three *a priori* ROIs for changes in healthy food craving, *B* = −0.08, 95% CI [−0.14, −0.02], SE = 0.03, *t*(124.50) = −2.84, *p* = 0.005, and only in the dlPFC ROI with regard to changes in the consumption of fruits and vegetables, *B* = −0.14, 95% CI [−0.27, −0.00], SE = 0.07, *t*(134.70) = −2.00, *p* = 0.047. Visual inspection of the healthy craving interaction across all three ROIs (Figure 4) indicated that people who recruited these brain regions more during regulation showed a decrease in their self-reported craving for healthy food over time, which was not seen in individuals who did not recruit these regulation network regions as strongly. Additional model results that did not pass the significance threshold are included in the Supplementary Material online.

## Discussion

The present study sought to investigate the associations between food craving reactivity and regulation-related brain activity and measures of craving for and consumption of healthy and unhealthy foods in a community sample of middle-aged adults with higher BMIs, as well as how this brain activity at baseline predicted changes in food craving and consumption over the course of 6 months.

### Baseline

#### Reactivity

Our analyses did not reveal any significant associations between food cue reactivity in the brain and self-reported liking of, craving for, or consumption of unhealthy—or healthy—food at baseline. This is surprising, given that the bulk of the brain-behavior research in this area has focused on food cue reactivity, and that a recent meta-analysis of this literature demonstrated a medium effect of food cue reactivity on food consumption and weight (Boswell and Kober, 2016). Because we controlled for BMI in our analyses in order to focus on brain-behavior associations, we may have obscured any weight-related effects. However, *post hoc* analyses showed that there was also no association between baseline BMI and reactivity-related brain activity in the present sample [*r*(88)-values = 0.024–0.087, *p* > 0.41]. As such, removing variance associated with BMI most likely did not obscure any weight-driven brain-behavior associations at baseline. It is also possible that these null results could be related to the contrast we used to operationalize reactivity in the brain. As evident in Figure 2A, there were very few clusters that differed significantly between the craved (LC) and not craved (LNC) foods, indicating that reactivity was similar across conditions, despite substantial differences in self-reported craving ratings for craved and not craved foods (Supplementary Table S1). Because this measure of cue reactivity does not appear to have been specific to craved foods here, it may account for not observing the expected relationships. Lastly, these null findings may also be due to limitations inherent to the self-report measures we used to assess food craving and consumption (e.g., self-presentation bias, imperfect memory recall). Accurate measurement of food consumption is notoriously challenging, and while the ASA24 retrospective food intake measure used in the present study is the measure of choice for the National Cancer Institute, a recent paper documented several usability challenges with this tool that may have led to reporting errors (Kupis et al., 2019).

#### Regulation

We also did not see any significant baseline associations between regulation-related activity and self-reported liking of, craving for, or consumption of unhealthy foods in our *a priori* ROIs. However, exploratory analyses revealed that regulation-related brain activity was positively associated with the craving for and consumption of healthy foods. Specifically, activity in an average of our three *a priori* ROIs (dlPFC, dACC, IFG) was significantly positively associated with fruit and vegetable consumption, which appeared to be driven by dlPFC activity. We also saw this effect with regard to healthy food craving in the vmPFC, and general healthy eating as well as fruit/vegetable intake in the left cerebellum and right supramarginal gyrus. We also found that right post-medial frontal activity was negatively associated with empty calorie consumption.

Together, these data demonstrate that greater engagement of these regions during the regulation of the desire for unhealthy food was associated with concurrent healthier eating. Activation in these regions could also be indexing health goals representations (Tusche and Hutcherson, 2018), motivation to change, or effort on and engagement with the task of down-regulating craving for unhealthy foods. While we expected to see this pattern in the traditional lateral prefrontal regions like the dlPFC, we were somewhat surprised that the cerebellum and supramarginal gyrus also showed such a consistent pattern of associations with healthy eating. These two regions are generally thought to underlie mentalizing and other types of social cognition (Van Overwalle and Mariën, 2016), yet some studies have found them to be significantly involved in cognitive control (e.g., Żurawska vel Grajewska et al., 2011). Therefore, it may be that individuals who engage these regions during regulation may be—consciously or subconsciously—using more socially-relevant regulation strategies, which may be associated with greater healthy food intake. While we have explored different types of craving regulation strategies in past work (see Giuliani et al., 2013), these have not yet included social strategies (Nook and Zaki, 2015).

The fact that almost all of the baseline associations we observed were in the domain of healthy eating also supports the assertion that our present lack of reactivity-consumption findings—which are traditionally seen in the domain of unhealthy food (e.g., Lopez et al., 2014)—may be due to the increased motivation of our participants to eat better. The specificity of the results to the healthy food domain may also be why we found relatively few effects on craving for these foods, as there are not as frequently craved as unhealthy foods (Massicotte et al., 2019). Interestingly, we also found no brain-behavior associations regarding the self-reported liking of healthy foods, which suggests that this pattern is specific to the motivational and ingestive aspects of eating behavior, and not the more subjective process of liking.

### Change Over Time

#### Reactivity

With regard to baseline levels of brain activity predicting overall changes in food liking, craving, and consumption over the course of the 6 month follow-up period, we did not find any evidence that brain activity during food cue reactivity predicted behavior changes in the domains of unhealthy or healthy foods. These results are contrary to much of the previous findings in this domain, which have shown relatively consistent associations between food cue reactivity and behavior change using dependent variables such as weight gain/loss, cue-induced eating, or snack consumption (e.g., Demos et al., 2012; Lawrence et al., 2012; Murdaugh et al., 2012; Versace et al., 2018). The present null result may be due to the paradigm we used to index food cue reactivity, which contrasted two energy-dense foods that only differed based on participant preference. This is quite different from most other studies in this domain, which usually contrast high energy-dense foods with low-density foods, if a contrasting condition is used at all. In addition, these results may be due to the sample, which consisted of community adults who were selected because they had BMIs between 25 and 40 but were also highly motivated to improve their eating habits. As such, the increased motivation of the subjects may have negated any hypothesized reactivity-consumption associations by influencing both brain reactivity to pictures of unhealthy foods and consumption of those foods.

#### Regulation

In contrast to the reactivity results, we did find that brain activity associated with regulating the desire for unhealthy foods was significantly associated with changes in craving for and consumption of healthy foods. Specifically, we found that increased regulation-related activity in the dlPFC, IFG, dACC, and vmPFC significantly predicted decreases in healthy food craving over time. Regulation-related activity in the dlPFC and vmPFC also predicted a decrease in self-reported fruit and vegetable consumption. Across all of these regions, greater engagement during regulation predicted less craving for and consumption of healthy foods over time. While the brain regions implicated here are those we expected would track with real-world regulation success, we found an opposite pattern than predicted: *less* brain activity during regulation was associated with *more* craving for and consumption of healthy foods.

This pattern of brain-as-predictor results is complex and somewhat contradictory, with more regulation-related activity associated with healthier eating at baseline, which then waned over the course of 6 months. One possible explanation is simply that craving regulation is multifaceted, reflecting many psychological processes including effort, attentional control, motivation, desire, meta-cognition and planning, among others. As such, simply asking people to indicate their food desire ratings via a button press after attempting to down-regulate their desire for a food may not get at the complexity of the process (e.g., effort, conflict, etc.). In addition, we used mean-level activation within ROIs as brain-based indices of reactivity and regulation, but because they are not specific indicators of these psychological processes, they likely represent integrated engagement of numerous cognitive functions. Future studies should investigate multivariate measures of reactivity and regulation to improve sensitivity and potentially predictive utility (Cosme et al., 2020). Regardless, the present findings suggest that the health neuroscience literature may benefit from an increased interrogation of the factors predicting healthy food consumption, instead of continuing to focus primarily on reducing the consumption of unhealthy food.

As mentioned earlier, some features of the sample itself are worth considering and might provide clues that help interpret the observed pattern of response. The sample consisted of community adults who were selected because they had higher BMIs, and also highly motivated to improve their eating habits. Indeed, it may be that this increased motivation to eat better upon study entry was reflected in both greater effort during regulation—which resulted in more brain activity—and healthier baseline eating behavior. Cognitive reappraisal, the regulation strategy employed in the present study, is thought to be relatively less effortful compared to more response-focused regulation strategies like suppression (Gross, 2002). However, this work was done using negative images; neural reactions to unhealthy food cues have been found to occur early and are relatively automatic (Meule et al., 2013). As such, reappraising the desire for a delicious-looking unhealthy food may actually be a form of late reappraisal, which has been shown to engage substantial inhibitory control resources (Sheppes et al., 2009). This level of effort is hard to maintain in the long run, which is why greater recruitment of these brain regions at baseline ultimately resulted in a downward trajectory of healthy eating over 6 months. These results suggest that targeting less effortful food craving regulation strategies may be more successful in helping people meet their healthy eating goals over longer periods of time. Recent work by Reader et al. (2018) supports this idea, showing that greater success at up-regulating the desire for healthy foods was associated with increased craving strength for low-calorie foods as well as decreased consumption of high-calorie foods in their daily lives.

### Limitations

The present findings should be interpreted in light of several limitations of the present study. First, the sample was majority female and Caucasian. We did employ broad recruitment in the community of a mid-sized city in the Pacific Northwest, United States, but the limited gender and racial makeup of the present sample may limit the generalizability of the present findings to other samples. In addition, the sample was entirely higher BMI, with no comparison group. As such, the patterns of results in this study may be reflective of this specific group, who may also process stimuli and approach food in ways different from lower-weight individuals. Second, while we excluded individuals who were actively enrolled in a diet program, we did not collect information about past diet attempts or weight fluctuations. Because previous studies have found an influence of dietary behavior on brain activity (e.g., Ely et al., 2014), this is an important caveat to the present results that should be addressed in future work. Third, while we worked hard to retain our sample throughout the full 6 month study protocol, we experienced substantial attrition, with only 52 of the original 88 participants providing BMI data at T4. A comparison of the participants who provided data throughout the study versus those who did not revealed that they did not differ significantly with regard to age, gender, or ethnicity. However, we cannot assess how this attrition may have impacted our longitudinal outcome measures, as we do not have follow-up data from participants who did not return to the lab at T4. Relatedly, participants reported that inventorying their 24-h food consumption using the ASA24 twice at each time point was cumbersome, which may have led to loss of follow-up data and perhaps also contributed to careless or more inaccurate reporting among those who did complete the follow-up assessments (Kupis et al., 2019). While accurately measuring actual food consumption is notoriously difficult, future work would benefit from employing a measure of food intake that participants are more likely to complete at multiple time points (i.e., taking photos of meals). Lastly, half of the participants in these analyses were enrolled in an intervention designed to increase their motivation to engage in healthier eating behaviors. While we collapsed across groups and included intervention condition as a covariate in all analyses, the fact that half of the participants had received this intervention between baseline and T2 may have affected the self-reported food craving and consumption data we collected at all three time points.

## Conclusion

Overall, this study demonstrated that brain activity associated with regulating desires for unhealthy food predicted meaningful changes in the craving for and consumption of healthy food over the course of 6 months in a population of middle-aged adults with higher BMIs. These nuanced findings add to the growing body of research on the neuroscience of eating, which has predominantly focused on the consumption of unhealthy foods and/or body weight, and highlights the importance of studying healthy eating behavior as well.

## Data Availability Statement

The datasets presented in this study can be found in online repositories. The names of the repository/repositories and accession number(s) can be found below: https://github.com/UOSAN/regulation-craving-consumption.

## Ethics Statement

The studies involving human participants were reviewed and approved by the Institutional Review Board of the University of Oregon. The patients/participants provided their written informed consent to participate in this study.

## Author Contributions

NG and EB designed the study. JM, BD, and NG collected the data. NG, JM, and DC analyzed the data. NG wrote the manuscript and created the figures. DC created the supplementary tables. DC, JM, BD, and EB provided edits. All authors contributed to the article and approved the submitted version.

## Conflict of Interest

EB was manager of Berkman Consultants, a boutique consulting firm specializing in goals, motivation, and behavior change. The remaining authors declare that the research was conducted in the absence of any commercial or financial relationships that could be construed as a potential conflict of interest.

## Footnotes

1 https://osf.io/uvrkj/

2 https://epi.grants.cancer.gov/asa24/resources/hei.html

3 http://www.fil.ion.ucl.ac.uk/spm

4 https://www.sas.upenn.edu/∼mcguirej/meta-analysis.html

5 https://github.com/UOSAN/regulation-craving-consumption
